# Prevalence and Factors Associated With Severe Obesity Among U.S. Adults: A Cross‐Sectional Study

**DOI:** 10.1155/jobe/3831008

**Published:** 2026-04-29

**Authors:** Nirajan Budhathoki, Joseph N. Inungu

**Affiliations:** ^1^ Henry Ford Health + Michigan State University Health Sciences, Detroit, Michigan, USA; ^2^ Department of Public Health Sciences, Henry Ford Health, Detroit, Michigan, USA, henryford.com; ^3^ School of Health Sciences, Central Michigan University, Mount Pleasant, Michigan, USA, cmich.edu

**Keywords:** adults, logistic regression, NHANES, sample weight, severe obesity, USA

## Abstract

**Background:**

Severe obesity (body mass index [BMI] of 40 kg/m^2^ or higher) poses significant health risks. It reflects a multifaceted interplay of genetic, behavioral, and environmental factors, contributing to rising rates of metabolic diseases and reduced quality of life. This study aimed to examine the prevalence and risk factors of severe obesity among adults in the United States.

**Methods:**

We analyzed data from the National Health and Nutrition Examination Survey (NHANES), a complex, multistage, cross‐sectional survey, designed to collect health and nutritional data from a representative sample of the civilian noninstitutionalized U.S. population. A total of 6729 respondents from the 2017 to March 2020 cycle of NHANES were included in the analysis. Descriptive statistics were used to summarize the characteristics of the sample. Multivariable logistic regression models were utilized to study risk factors for severe obesity. All analyses accounted for the complex survey design of the NHANES.

**Results:**

The weighted prevalence of severe obesity among adults was 9.4%, with a higher rate found in females, 11.9%, compared to males, 6.2%. Binary logistic regression analysis revealed that males had 39% lower odds of severe obesity compared to females (odds ratio [OR]: 0.61, 95% confidence interval [CI]: 0.32–1.14), though this was not statistically significant. Participants aged 31–40 years were more than twice as likely to have severe obesity compared to those aged 20–30 years (OR: 2.73, 95% CI: 1.07–6.96). Non‐Hispanic Asians had significantly lower odds of severe obesity than Mexican Americans (OR: 0.06, 95% CI: 0.01–0.27). Arthritis nearly doubled the odds of severe obesity (OR: 1.94, 95% CI: 1.17–3.22), and high blood pressure showed the strongest relationship, increasing odds more than fourfold (OR: 4.42, 95% CI: 3.30–5.91). Marital status, health insurance, and income level showed no significant associations with severe obesity. These findings highlight the complex interplay of factors influencing severe obesity.

**Conclusion:**

In conclusion, the study highlights a significant prevalence of severe obesity among adults in the United States, particularly among females, individuals aged 31–40 years, and those with specific comorbid conditions such as high blood pressure and arthritis. Non‐Hispanic Asians exhibited notably lower odds of severe obesity compared to other racial/ethnic groups, underscoring potential protective factors. The findings emphasize the multifactorial nature of severe obesity, driven by demographic, behavioral, and health‐related factors, and underline the importance of tailored interventions to address this public health challenge.

## 1. Background

Obesity is a major public health epidemic, with over one billion adults being overweight and 300 million obese worldwide [1]. The World Health Organization (WHO) defines obesity as a chronic disease characterized by abnormal or excessive fat accumulation usually caused by an imbalance between calories consumed and expended [2]. Projections estimate over 2.16 billion overweight and 1.12 billion obese individuals globally by 2030 [3]. Body mass index (BMI) is the most commonly used measure of weight. A BMI of 30 kg/m^2^ or higher is classified as obese, whereas a BMI of 40 kg/m^2^ or higher is referred to as Class 3 or severe obesity [4].

In the United States, the prevalence of obesity among adults has reached 42.4%, with severe obesity at 9.2% in 2017–2018, reflecting a steady increase from 1999 to 2000 [5]. Obesity is associated with elevated risks of coronary heart disease [6], end‐stage renal disease [7], stroke [8], type 2 diabetes [9], and certain types of cancer [10]. Furthermore, severe obesity is an independent risk factor for COVID‐19 mortality among hospitalized patients aged < 50 years [11]. The economic burden is also substantial, with medical costs for individuals with obesity exceeding those for individuals with a healthy weight by more than $1800 annually [12]. This dual impact on health and economic systems underscores the urgency of addressing obesity—particularly severe obesity—as a critical public health priority [13].

The mechanisms underlying obesity are complex and involve a dynamic interplay of genetic, environmental, socioeconomic, and individual behavioral factors [14–16]. Genetic predisposition influences an individual’s susceptibility to weight gain [16], while environmental factors such as sedentary lifestyles, widespread availability and marketing of energy‐dense foods, and urbanization have significantly contributed to the global rise in obesity prevalence [17]. Socioeconomic status is another critical determinant, with obesity disproportionately affecting individuals with lower socioeconomic status, particularly in developing countries [16].

A substantial body of research has examined the risk factors associated with overweight and obesity [18, 19]; however, studies specifically addressing severe obesity remain limited [20]. This study aimed to fill this gap by identifying the key factors associated with severe obesity among U.S. adults. A comprehensive understanding of these factors could lead to the development of targeted interventions to alleviate the increasing global burden of severe obesity.

## 2. Methods

### 2.1. Data Source

**FIGURE 1:**
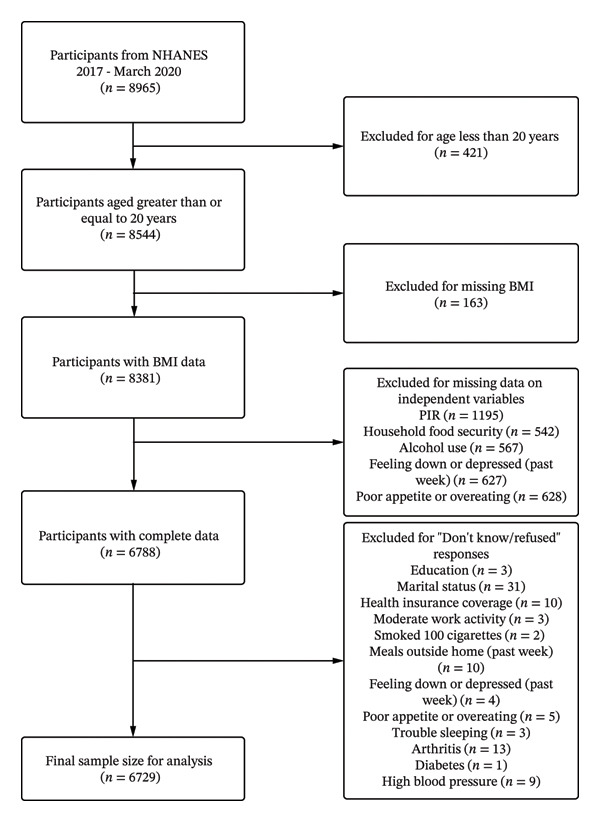
Flow diagram for participant selection in the study.

Data were taken from the 2017 to March 2020 cycle of the National Health and Nutrition Examination Survey (NHANES). NHANES is a complex, multistage, cross‐sectional survey designed to collect health and nutritional data from a representative sample of the civilian noninstitutionalized U.S. population. The design and operation of NHANES have been described previously [21]. The survey is conducted by a diverse team comprising health surveyors, medical technicians, and physicians. It generates an extensive database that includes demographic details, dietary patterns, clinical assessments, laboratory findings, and responses to structured questionnaires. This study followed the Strengthening the Reporting of Observational Studies in Epidemiology (STROBE) reporting guideline for cross‐sectional studies (Supporting Table S1). A total of 6729 survey participants aged 20 years or more with complete data were included in the analysis. Figure 1 presents the flow diagram for participant selection in this study.

### 2.2. Outcome Variable

“Severe Obesity” was our outcome of interest. Survey participants with BMI values of 40 kg/m^2^ or greater were classified as severe obesity cases, while those in the BMI range of 18.5–24.9 kg/m^2^ were classified as normal.

### 2.3. Independent Variables

Based on existing literature and clinical considerations [22, 23], this study included several confounding factors, including (1) Demographic and socio‐economic variables: age, sex, race, education, marital status, health insurance coverage, household food security, and poverty‐to‐income ratio (PIR); (2) Personal habits: smoking, alcohol consumption, and moderate work activity; (3) Dietary habits: number of meals not prepared at home (in the past 7 days), poor appetite or overeating; and (4) Medical conditions: self‐reported feeling down or depressed, trouble sleeping, arthritis, diabetes, and high blood pressure.

Although the respondent’s age was reported in actual years, we categorized it into six levels: 20–30 years, 31–40 years, 41–50 years, 51–60 years, 61–70 years, and more than 70 years. Race/ethnicity was categorized as follows: Non‐Hispanic White, Non‐Hispanic Black, Non‐Hispanic Asian, Mexican American, Other Hispanic, and Other Race. Education level was classified into three categories: less than high school, high school graduate, and some college or more.

NHANES records respondents’ household food security level for the last 12 months of the survey in four categories: full, marginal, low, and very low. To derive those categories, NHANES uses responses on 18 items for households with children under the age of 18 years and 10 items for households without children. A detailed description of the items used and the corresponding four categories can be found in the NHANES official documentation, for which a link has been provided previously. Economic status was assessed via PIR, which was collected as a continuous measure ranging from 0 to 5 (for values greater than or equal to 5). Following [24], we re‐leveled this ratio into two categories: income below poverty line: PIR ≤ 1 and income above poverty line: PIR > 1. With regard to physical activity, respondents answered “Yes” or “No” to the question, “Does your work involve moderate‐intensity activity that causes small increases in breathing or heart rate such as brisk walking or carrying light loads for at least 10 min continuously?” Respondents’ smoking behavior was assessed based on the response to the question “Have you smoked at least 100 cigarettes in your entire life?” With regard to alcohol use, respondents were asked, “In your entire life, have you had at least 1 drink of any kind of alcohol, not counting small tastes or sips?”

To gather data on dietary habits, respondents were asked, “During the past 7 days, how many meals did you get that were prepared away from home in places such as restaurants, fast food places, food stands, grocery stores, or from vending machines?” We categorized the responses to this question into four levels: none, up to 3, 4 to 7, and more than 7. Additionally, their dietary habit was surveyed with the question “Over the last 2 weeks, how often have you been bothered by the following problems: poor appetite or overeating?” To assess sleeping behavior, respondents were asked, “Have you ever told a doctor or other health professional that you have trouble sleeping?” Individuals were considered to have diabetes if they responded, “Yes” to the question, “Other than during pregnancy, have you ever been told by a doctor or health professional that you have diabetes or sugar diabetes?” Regarding arthritis status, respondents were asked, “Has a doctor or other health professional ever told you that you had arthritis?” Finally, information on high blood pressure was collected via the survey question, “Have you ever been told by a doctor or other health professional that you had hypertension, also called high blood pressure?”

### 2.4. Statistical Analysis

Descriptive statistics, including frequency, proportions, and the chi‐square test of association, were used to describe the study population. This was followed by univariable and multivariable analyses using logistic regression to study associations between the outcome variable “Severe Obesity” and independent variables. Variables statistically significantly associated with the outcome in the bivariate analysis were included in the multivariable model. The multivariable logistic regression was used to simultaneously adjust for the independent variables in the final model. We reported both unadjusted odds ratio (OR) and adjusted odds ratio (aOR) with their corresponding 95% confidence intervals (CIs). To be able to generalize the study findings to the entire U.S. population, weighted analyses were conducted using the R [25] *survey* [26] package with CDC‐computed study‐specific weights provided for each respondent. The weighted analyses are important to account for the fact that NHANES uses a complex, multistage probability sampling design to select survey participants. Collinearity among independent variables was assessed by estimating the variance inflation factor (VIF) with the *svydiags* package in R, which is built specifically for survey datasets [27]. A complete case analysis that excluded responses with missing values was performed. Two‐tailed *p* < 0.05 was considered statistically significant.

## 3. Results

Table 1 presents the characteristics of the study participants classified into four BMI classes: severely obese, obese or overweight, normal, and underweight. The sex distribution revealed a higher proportion of severely obese females compared to males (11.9% vs. 6.2%). Severe obesity was most prevalent in the 31–40 years age group (13.1%) and least prevalent in the > 70 years age group (4.6%). Racial differences were evident, with the non‐Hispanic Black population having the highest proportion of severe obesity (13.6%). Conversely, the non‐Hispanic Asian population had only 1.6% severely obese. Concerning education level, 10.4% of high school graduates were severely obese. Additionally, 11.5% of single participants were classified as severely obese compared to 8.7% reported among those married or living with a partner. There was no significant difference in the proportion of severe obesity between participants with and without health insurance coverage (9.7% vs. 9.1%). Among participants with very low food security, 14.6% were severely obese, while those with full food security had the highest proportion (8%) classified as obese or overweight. Severe obesity prevalence also varied by income status, with participants below the poverty line having a higher prevalence (10.6%) than those above the poverty line (9%).

**TABLE 1:** NHANES 2017–March 2020 respondent characteristics by body mass index (BMI) classes (*N* = 6729).

	Severely obese		Obese or overweight		Normal		Underweight		*p* value^3^
	*N* ^1^	Weighted%^2^	*N* ^1^	Weighted%^2^	*N* ^1^	Weighted%^2^	*N* ^1^	Weighted%^2^	
Gender									
Female	448	11.9	2107	59	846	27.2	49	1.8	< 0.01
Male	213	6.2	2279	71.3	750	21.7	37	0.8	
Age group									
20–30	98	7.6	594	52.6	395	36.7	34	3.1	< 0.01
31–40	137	13.1	647	61.8	244	24.2	13	0.9	
41–50	134	10.8	723	67.5	218	21.2	7	0.5	
51–60	149	10.7	827	67.0	254	20.3	16	2	
61–70	101	6.7	887	71.8	250	21.2	8	0.4	
More than 70	42	4.6	708	74.1	235	20.8	8	0.5	
Race									
Mexican American	62	7.7	572	77.1	110	14.4	4	0.8	< 0.01
Non‐Hispanic Asian	10	1.6	384	51.2	349	45.7	10	1.6	
Non‐Hispanic Black	251	13.6	1110	64.1	351	20.8	26	1.5	
Non‐Hispanic White	257	9.3	1614	63.7	599	25.5	39	1.4	
Other Hispanic and other race	81	8.6	706	70.9	187	19.7	7	0.8	
Education									
Below high school	91	8.5	763	68.3	234	21.6	15	1.6	< 0.01
High school graduate	183	10.4	1037	65.7	369	21.7	32	2.1	
Some college or more	387	8.8	2586	64.1	993	26.2	39	0.9	
Marital status									
Married/living with partner	343	8.7	2689	68.5	881	21.5	43	1.2	< 0.01
Never married	170	11.5	684	51.3	386	34.7	28	2.5	
Widowed/divorced/separated	148	8.6	1013	66.3	329	24.7	15	0.5	
Health insurance coverage									
Yes	556	9.1	3740	65.3	1339	24.4	66	1.2	0.053
No	105	9.7	646	62.1	257	25.8	20	2.4	
Household food security									
Full food security	351	8	2811	65.8	1062	25.1	51	1.1	0.017
Marginal food security	104	11.2	606	64.3	197	22.7	15	1.8	
Low food security	118	12.1	592	63.9	187	21.7	13	2.3	
Very low food security	88	14.6	377	58.7	150	25.6	7	1.1	
PIR									
Income above poverty line	524	9	3595	65.4	1288	24.5	57	1.1	< 0.01
Income below poverty line	137	10.6	791	61.7	308	24.5	29	3.2	
Moderate work activity									
Yes	318	9.2	2012	64.6	680	25	36	1.2	0.829
No	343	9.2	2374	65.3	916	24.1	50	1.5	
Smoked 100 cigarettes									
Yes	294	10.2	1876	64.9	651	23.5	48	1.4	0.321
No	367	8.4	2510	65	945	25.3	38	1.3	
Alcohol use									
Yes	622	9.4	4027	65	1432	24.3	78	1.3	0.114
No	39	6.4	359	64	164	28.4	8	1.1	
Meals outside home (past week)									
More than 7	57	9.5	412	63.8	156	26.2	4	0.5	0.174
4 to 7	185	10.8	1032	65.5	345	22.8	19	1	
Up to 3	321	9.1	2100	65.5	738	23.9	46	1.5	
None	98	6.3	842	63.1	357	28.6	17	2	
Felt down or depressed (past week)									
Half the days or more	57	9.7	342	63.5	95	23.1	11	3.7	0.04
Several days	143	11.4	765	62.2	272	24.7	23	1.7	
Not at all	461	8.6	3279	65.7	1229	24.6	52	1.1	
Poor appetite or overeating									
Half the days or more	119	18.5	419	58.8	119	21.3	12	1.5	< 0.01
Several days	150	14.4	683	65.3	203	18.9	13	1.4	
Not at all	392	7.1	3284	65.6	1274	26.1	61	1.3	
Trouble sleeping									
Yes	278	13.1	1331	65.2	361	20.5	20	1.2	< 0.01
No	383	7.4	3055	64.8	1235	26.4	66	1.4	
Arthritis									
Yes	283	12.9	1405	68.6	341	17.7	16	0.7	< 0.01
No	378	7.7	2981	63.5	1255	27.2	70	1.6	
Diabetes									
Yes	157	16.5	743	74	122	9.3	2	0.3	< 0.01
Borderline	24	21.3	124	57.6	43	15.8	2	5.3	
No	480	7.9	3519	63.9	1431	26.8	82	1.4	
High blood pressure									
Yes	341	13.2	1852	73.1	374	13.4	14	0.4	< 0.01
No	320	7.3	2534	61	1222	29.9	72	1.8	

*Note:* Overall weighted prevalence of severe obesity was 9.4%.

^1^Raw frequency.

^2^Computed using CDC‐provided weights for each respondent.

^3^Obtained from Pearson’s chi‐square test with Rao and Scott adjustment.

No statistically significant associations were observed between BMI classes and moderate work activity (*p* = 0.829), smoking at least 100 cigarettes in a lifetime (*p* = 0.321), alcohol consumption (*p* = 0.114), or the number of meals eaten outside the home during the week before the survey (*p* = 0.174). Among participants who felt down or depressed for several days in the past week, 11.4% were severely obese, and 62.2% were obese or overweight. Participants who experienced poor appetite or overeating for half the days or more had the highest proportion in the severe obesity category (18.5%) and the lowest in the underweight category (1.5%). Participants reporting trouble sleeping had a higher prevalence of severe obesity (13.1%) compared to those without trouble sleeping (7.4%). Severe obesity was more prevalent among participants with arthritis (12.9%), borderline diabetes (21.3%), and high blood pressure (13.2%) compared to those without these conditions.

Table 2 presents the results of the univariable logistic regression modeling severe obesity. It may be observed that males have significantly lower odds of severe obesity than females (OR: 0.66, 95% CI: 0.47–0.93). Concerning age group, middle‐aged adults (31–60 years) have higher odds of severe obesity compared to the reference group (20–30 years). The odds of severe obesity were significantly higher for age groups 31–40 (OR: 2.60, 95% CI: 1.71–3.97), 41–50 (OR: 2.45, 95% CI: 1.59–3.78), and 51–60 (OR: 2.54, 95% CI: 1.56–4.13) than the reference age group (20–30 years). Older age groups (61 years and above) show reduced and statistically nonsignificant associations. Non‐Hispanic Asians have markedly lower odds of severe obesity (OR: 0.07, 95% CI: 0.03–0.16) compared to Mexican Americans. No statistically significant differences were observed in the odds of severe obesity across different levels of education, marital status, health insurance coverage, PIR, moderate work activity, and feeling down or depressed.

**TABLE 2:** Univariable logistic regression results to study odds of severe obesity compared to the normal BMI category.

Variables	Unadjusted odds ratio	95% CI	
		Lower bound	Upper bound
Gender			
Female	Reference		
Male	**0.66**	**0.47**	**0.93**
Age group			
20–30	Reference		
31–40	**2.60**	**1.71**	**3.97**
41–50	**2.45**	**1.59**	**3.78**
51–60	**2.54**	**1.56**	**4.13**
61–70	1.51	0.94	2.43
More than 70	1.07	0.58	1.99
Race			
Mexican American	Reference		
Non‐Hispanic Asian	**0.07**	**0.03**	**0.16**
Non‐Hispanic Black	1.23	0.71	2.13
Non‐Hispanic White	0.69	0.39	1.22
Other Hispanic and other race	0.82	0.43	1.57
Education			
Below high school	Reference		
High school graduate	1.22	0.86	1.72
Some college or more	0.85	0.61	1.19
Marital status			
Married/living with partner	Reference		
Never married	0.82	0.53	1.27
Widowed/divorced/separated	0.86	0.59	1.26
Health insurance coverage			
Yes	1.00	0.73	1.36
No	Reference		
Household food security			
Full food security	Reference		
Marginal food security	1.54	0.95	2.49
Low food security	**1.73**	**1.19**	**2.53**
Very low food security	**1.79**	**1.34**	**2.38**
PIR			
Income above poverty line	Reference		
Income below poverty line	1.18	0.79	1.76
Moderate work activity			
Yes	0.96	0.76	1.21
No	Reference		
Smoked 100 cigarettes			
Yes	**1.30**	**1.02**	**1.66**
No	Reference		
Alcohol use			
Yes	**1.70**	**1.08**	**2.68**
No	Reference		
Meals outside home (past week)			
More than 7	1.65	0.79	3.47
4 to 7	**2.16**	**1.44**	**3.25**
Up to 3	**1.74**	**1.22**	**2.49**
None	Reference		
Felt down or depressed (past week)			
Half the days or more	Reference		
Several days	1.11	0.62	1.98
Not at all	0.84	0.49	1.43
Poor appetite or overeating			
Half the days or more	Reference		
Several days	0.88	0.56	1.38
Not at all	**0.31**	**0.21**	**0.46**
Trouble sleeping			
Yes	**2.27**	**1.69**	**3.06**
No	Reference		
Arthritis			
Yes	**2.57**	**1.83**	**3.61**
No	Reference		
Diabetes			
Yes	1.32	0.62	2.80
Borderline	Reference		
No	**0.22**	**0.09**	**0.54**
High blood pressure			
Yes	**4.06**	**3.29**	**5.02**
No	Reference		

*Note:* Statistically significant results are printed in bold.

Lower food security is associated with increased odds of severe obesity. Specifically, low food security (OR: 1.73, 95% CI: 1.19–2.53) and very low food security (OR: 1.79, 95% CI: 1.34–2.38) were associated with increased odds of severe obesity compared to those with full food security. Participants who smoked and consumed alcohol had significantly higher odds of severe obesity (OR: 1.30, 95% CI: 1.02–1.66) and (OR: 1.70, 95% CI: 1.08–2.68), respectively. This univariate analysis found that more meals consumed outside the home were associated with higher odds of severe obesity. In particular, those who ate up to three meals outside the home during a week had 74% higher odds (OR: 1.74, 95% CI: 1.22–2.49), and those who ate four to seven meals outside had 116% higher odds (OR: 2.16, 95% CI: 1.44–3.25) than those who ate no meals outside the home. Those who did not have self‐reported poor appetite or overeating concerns had lower odds (OR: 0.31, 95% CI: 0.21–0.46) compared to those who had such concerns half the days or more during the week before the survey. Respondents without diabetes had reduced odds of severe obesity (OR: 0.22, 95% CI: 0.09–0.54). Respondents who reported having trouble sleeping (OR: 2.27, 95% CI: 1.69–3.06), arthritis (OR: 2.57, 95% CI: 1.83–3.61), and high blood pressure (OR: 4.06, 95% CI: 3.29–5.02) had significantly higher odds of severe obesity than those without the conditions. These results highlight the impact of each independent variable on the odds of severe obesity, without adjusting for the effects of other variables in the model. To summarize, Table 2 shows that factors such as middle age, low food security, lifestyle behaviors, and comorbid conditions (e.g., high blood pressure and arthritis) are strong predictors.

Table 3 presents the binary logistic regression analysis results assessing the odds of severe obesity while controlling for various demographic, socioeconomic, and health‐related factors. The findings revealed that males had 39% lower odds of developing severe obesity than females (OR: 0.61; 95% CI: 0.32–1.14), although this result was not statistically significant. Participants aged 31–40 years were more than twice as likely to have severe obesity compared to those aged 20–30 years (OR: 2.73; 95% CI: 1.07–6.96); the difference was statistically significant.

**TABLE 3:** Multivariable logistic regression results to study odds of severe obesity compared to the normal BMI category.

Variables	Adjusted odds ratio	95% CI	
		Lower bound	Upper bound
Gender			
Female	Reference		
Male	0.61	0.32	1.14
Age group			
20–30	Reference		
31–40	**2.73**	**1.07**	**6.96**
41–50	2.07	0.79	5.41
51–60	1.39	0.49	3.96
61–70	0.79	0.23	2.66
More than 70	0.40	0.11	1.53
Race			
Mexican American	Reference		
Non‐Hispanic Asian	**0.06**	**0.01**	**0.27**
Non‐Hispanic Black	0.95	0.38	2.36
Non‐Hispanic White	0.55	0.19	1.55
Other Hispanic and other race	0.7	0.27	1.85
Education			
Below high school	Reference		
High school graduate	1.19	0.69	2.05
Some college or more	0.89	0.53	1.47
Marital status			
Married/living with partner	Reference		
Never married	1.12	0.46	2.75
Widowed/divorced/separated	0.62	0.32	1.17
Household food security			
Full food security	Reference		
Marginal food security	1.25	0.58	2.72
Low food security	1.37	0.73	2.58
Very low food security	1.33	0.69	2.57
Health insurance coverage			
Yes	Reference		
No	0.97	0.42	2.26
PIR			
Income above poverty line	Reference		
Income below poverty line	0.75	0.36	1.56
Trouble sleeping			
Yes	1.59	0.93	2.71
No	Reference		
Arthritis			
Yes	**1.94**	**1.17**	**3.22**
No	Reference		
High blood pressure			
Yes	**4.42**	**3.30**	**5.91**
No	Reference		

*Note:* Statistically significant results are printed in bold.

Non‐Hispanic Asians were significantly less likely to have severe obesity compared to Mexican Americans (OR: 0.06; 95% CI: 0.01–0.27). Widowed/divorced/separated individuals had 38% lower odds of severe obesity compared to those who were married or living with a partner (OR: 0.62; 95% CI: 0.32–1.17), but this association was not statistically significant. Similarly, having health insurance (OR: 0.97, 95% CI, 0.42–2.26) or an income below the poverty line (OR: 0.75, 95% CI, 0.36–1.56) had reduced but statistically insignificant odds of severe obesity.

Self‐reported trouble sleeping was associated with a 59% increase in the odds of severe obesity (OR: 1.59; 95% CI: 0.93–2.71), though this finding did not achieve statistical significance. Comorbid conditions demonstrated a stronger relationship with severe obesity. Arthritis was associated with significantly higher odds of the condition (OR: 1.94; 95% CI: 1.17–3.22). High blood pressure exhibited the strongest association, with affected individuals having more than four times the odds of severe obesity compared to those without high blood pressure (OR: 4.42; 95% CI: 3.30–5.91), a highly significant finding.

## 4. Discussion

This study aimed to investigate the prevalence and determinants of severe obesity among adults in the United States. Our study found that men were less likely than women to develop severe obesity, although the difference was not statistically significant. Studies consistently show that women are more likely to experience obesity and severe obesity than men, with global prevalence rates higher among females [28]. This disparity is attributed, in part, to differences in fat distribution, hormonal influences such as the effects of estrogen, and cultural factors that impact activity levels and dietary habits. Factors that contribute to this sex difference include a higher susceptibility to weight gain among women, particularly following major life events like pregnancy and menopause, as well as sociocultural norms that place greater pressure on women to conform to unrealistic beauty ideals [29]. These differences highlight the need for tailored interventions that account for physiological and cultural influences on obesity development and outcomes.

This study revealed a nonlinear, age‐dependent relationship between severe obesity and age: Adults aged 31–40 years had nearly three times the odds of severe obesity compared to those aged 20–30 years, but this excess risk diminished and became nonsignificant in the 41–50 and 51–60‐year age groups. The peak in early to mid‐adulthood likely reflects lifestyle changes, decreased physical activity, and unhealthy dietary patterns typical of this life stage [5, 30], while the subsequent decline may result from metabolic adaptations, increased health awareness, and alterations in body composition as one ages [31, 32]. Notably, a further rise in obesity is not observed among the “oldest‐old” (≥ 85 years), despite continued reductions in basal metabolic rate. This is because age‐related anorexia, frailty‐induced catabolism, sarcopenia, survivor bias, and restrictive living circumstances collectively limit net weight gain [33, 34]. These findings underscore the complex interplay of physiological, behavioral, and survival factors that shape obesity trajectories throughout the lifespan, highlighting the need for longitudinal studies to elucidate the underlying mechanisms.

The association between race and severe obesity reflects significant disparities influenced by genetic, cultural, behavioral, and socioeconomic factors. Non‐Hispanic Asians consistently show lower odds of both obesity and severe obesity compared to other racial and ethnic groups [35]. Several studies attribute this difference to lower average BMI and body fat percentage thresholds among Asians due to genetic predispositions for leaner body compositions [35]. Additionally, traditional dietary patterns in many Asian cultures, which emphasize whole grains, vegetables, and lower‐fat protein sources, contribute to reduced obesity risks [36]. Higher physical activity levels in some Asian populations may also play a protective role. Socioeconomic and environmental factors, including access to healthy food and healthcare, also influence these observed disparities. Such findings underscore the need for race‐specific approaches to obesity prevention and intervention [37].

Poor sleep is strongly associated with an increased risk of severe obesity [38]. Insufficient sleep duration and poor sleep quality disrupt metabolic processes, promoting weight gain. Sleep deprivation is linked to hormonal imbalances, including increased ghrelin (hunger hormone) and decreased leptin (satiety hormone), leading to greater appetite and caloric intake [39]. Furthermore, individuals with short sleep duration are more likely to engage in sedentary behaviors and consume calorie‐dense foods [40], both of which contribute to obesity. For example, one study found that individuals with shorter sleep durations had significantly higher odds of being obese (OR: 1.75) after adjusting for confounders such as age and race. Additionally, sleep disturbances, such as those caused by obstructive sleep apnea, exacerbate obesity by increasing systemic inflammation and altering circadian rhythms. These disruptions may further increase visceral fat accumulation and metabolic dysfunction, compounding the risk of severe obesity [39]. Addressing sleep health as part of obesity management could yield significant benefits.

The association between arthritis and severe obesity is well‐documented, as obesity significantly increases the risk of developing arthritis, particularly osteoarthritis [41]. Excess body weight places additional mechanical stress on weight‐bearing joints, such as the knees and hips, leading to accelerated cartilage degeneration and joint damage. Beyond mechanical factors, severe obesity contributes to a systemic inflammatory state, characterized by elevated levels of proinflammatory cytokines such as interleukin‐6 and tumor necrosis factor‐alpha. This chronic inflammation exacerbates joint inflammation and pain, further worsening arthritis symptoms [23]. Moreover, individuals with severe obesity often experience reduced mobility, which can lead to muscle weakness and further joint instability, compounding the burden of arthritis. These intertwined mechanisms highlight the bidirectional relationship, where arthritis‐related pain and functional limitations may also hinder physical activity, perpetuating the cycle of obesity and worsening arthritis outcomes [23].

Our findings indicate that individuals with high blood pressure have elevated odds of being severely obese. Future weight gain has been shown to be significantly greater in hypertensive subjects than in normotensive subjects in a previous study by [42]. It is important to note that prior studies have also established obesity‐induced hypertension, indicating the relationship between obesity and hypertension as a “two‐way street” [43, 44]. Our study, by no means, attempted to make a causal statement to infer which of the two conditions caused the other. Further studies are required to investigate the directionality of the association between these two conditions. Nevertheless, due to the rising rates of arthritis and hypertension among adults in the U.S. [45, 46], clinicians should screen for these conditions to evaluate the risk of severe obesity.

The strength of this study lies in its use of a nationally representative sample of adults. Using survey weights in all analyses enabled the findings to be generalized to the entire U.S. adult population. Important limitations include self‐reported data from the survey respondents, which may be susceptible to bias. The study’s cross‐sectional design prevents the establishment of causal relationships; however, it provides a basis for generating hypotheses for further research.

## 5. Conclusions

This study underscores the significance of several demographic and health characteristics associated with severe obesity among U.S. adults, in both males and females. While sex itself is not a modifiable risk factor, understanding the mechanisms by which females disproportionately develop severe obesity is crucial. Recognizing these disparities is the key to the development of targeted interventions. Future strategies should focus on at‐risk demographics and aim to modify the factors identified in this study to combat severe obesity. Finally, longitudinal studies would help to clarify the causal pathways between severe obesity and associated comorbidities such as hypertension and arthritis.

## Author Contributions

Nirajan Budhathoki conceptualized the project, performed the analysis, and drafted the manuscript. Nirajan Budhathoki and Joseph N. Inungu developed the methodology and reviewed data analysis. Nirajan Budhathoki and Joseph N. Inungu reviewed and edited the final manuscript.

## Funding

This research received no external funding.

## Disclosure

All the authors approved the final manuscript.

## Ethics Statement

NHANES was conducted with an approved protocol from the National Center for Health Statistics (NCHS) Ethics Review Board, Protocol Number: NHANES 2017–2018 (Continuation of Protocol #2011‐17, Protocol #2018‐01); NHANES 2019–2020 (Protocol #2018‐01). Informed consent was obtained from all survey participants.

## Consent

Please see the ethics statement.

## Conflicts of Interest

The authors declare no conflicts of interest.

## Supporting Information

Supporting Information 1. The supporting table (S1) is provided to demonstrate that the study was conducted following the Strengthening the Reporting of Observational Studies in Epidemiology (STROBE) reporting guideline for cross‐sectional studies.

## Supporting information


**Supporting Information** Additional supporting information can be found online in the Supporting Information section.

## Data Availability

Data used in the study are publicly available from the NHANES database at https://www.cdc.gov/nchs/nhanes/index.html.
